# The Effects of Destruxin A on Relish and Rel Gene Regulation to the Suspected Immune-Related Genes of Silkworm

**DOI:** 10.3390/molecules22010041

**Published:** 2016-12-29

**Authors:** Weina Hu, Guangwei He, Jingjing Wang, Qiongbo Hu

**Affiliations:** Key Laboratory of Bio-Pesticide Innovation and Application of Guangdong Province, College of Agriculture, South China Agricultural University, Guangzhou 510642, China; hwn688094@163.com (W.H.); a1020574494@126.com (G.H.); wangjingjing968@126.com (J.W.)

**Keywords:** destruxin, Bm12 cell, Imd, TollL, Relish, Rel

## Abstract

Destruxin A (DA), a cyclodepsipeptidic mycotoxin of entomopathogenic fungus, *Metarhizium anisopliae*, has anti-immunity activity against insects, but the mechanism of immune regulation is not clear yet. In our previous experiment, the significant expression changes of *Bm_nscaf2838_045*, *Bm_nscaf2674_066*, and *Bm_nscaf2767_133* genes in a silkworm’s hemocytes were found, which suggested that these genes might be involved in insect’s innate immunity. In the current experiment, the silkworm cell line Bm12 was used to survey the expression levels of these genes after the cells were treated with DA and the transcription factors *BmRel*, *BmRelish1* and *BmRelish2* were silenced by specific siRNA. The results indicated that, after the cells were treated by DA, the gene expression level of *BmRelish2* was significantly downregulated, but *BmRel* and *BmRelish1* were not changed. The results also showed that the gene expression levels of *Bm_nscaf2838_045* and *Bm_nscaf2674_066* had similar phenomena, i.e., downregulation with individual *BmRelish1* gene silence or DA treatment, upregulation with combination of *BmRelish1* gene silence and DA treatment, upregulation with individual *BmRelish2* gene silence, and downregulation with combination of *BmRelish2* gene silence plus DA treatment, but no changes in the *BmRel* gene silence combined with DA treatment. For the *Bm_nscaf2767_133* gene, the downregulated expressions were found in individual *BmRelish2* gene silence or DA treatment, upregulation in the combination treatment of *BmRelish2* gene silence plus DA, and the individual treatment of *BmRel* or *BmRelish1* silence. It is suggested that expressions of the *Bm_nscaf2838_045* and *Bm_nscaf2674_066* genes are closely related to the Imd signal pathway, but *Bm_nscaf2767_133* genes might involve in both Toll and Imd pathways. Furthermore, the *BmRelish1* gene acts as an activator and the *BmRelish2* gene acts as a repressor for both *Bm_nscaf2838_045* and *Bm_nscaf2674_066* gene expressions. It also implies that DA may participate in the splicing process of *BmRelish* where *BmRelish2* was promoted. Our research will provide new insights on the understanding of the activity mechanisms of destruxins.

## 1. Introduction

Destruxins are cyclodepsipeptidic mycotoxins with 39 analogues. Destruxin A (DA), the common analogue of entomopathogenic fungus, *Metarhizium anisopliae*, has substantial insecticidal activity [1,2,3]. Research has shown that DA can destroy an insect’s innate immune system, which includes breaking the balance between calcium ion and hydrogen ion in hemocytes [4], affecting the function of phagocytosis and encapsulation in hemocytes [5] and inhibiting the biosynthesis of the antibacterial peptides in drosophila [6]. Our previous research suggests that, although relatively large doses of DA is required to kill the silkworm (*Bombyx mori*) Bm12 cell, smaller dosages are sufficient to cause morphological changes [7]. We also found that the expression levels of the *Bm_nscaf2838_045* (NCBI GeneBank access NO. KY379951), *Bm_nscaf2674_066* (NCBI GeneBank access NO. KY379952), and *Bm_nscaf2767_133* (NCBI GeneBank access NO. KY379953) genes in silkworm’s hemocytes were influenced after DA treatment. Analyzing the gene expression profile showed that these genes were characterized as a cuticular protein gene or a basophilic serine protease gene [8]. However, the interaction mechanism of DA with these genes is not clear.

Generally, the Rel/NF-κB signal pathway plays an important role in insect immunization. Toll and Imd pathways belong to the Rel/NF-κB signal pathway. The insect’s immune reaction against fungi and Gram-positive bacteria is activated through the Toll signal pathway and against Gram-negative bacteria through the Imd signal pathway. Rel protein is a downstream transcription factor in the Toll pathway, but Relish protein is a transcription factor of the Imd pathway. The activated transcription factors enter cell nucleus and start to transcribe the target genes of antimicrobial peptides [9,10]. Silkworm has two Rel proteins, namely, Dorsal isoforms A and B, which were respectively coded by *BmRelA* and *BmRelB* transcripts from the selectively spliced *BmRel* gene [11,12]. *BmRelish1* and *BmRelish2*, the two transcripts of the *BmRelish* genes of *B. mori,* respectively code two proteins, NF-κB p110 subunit isoforms 1 and 2 [11,12]. However, it is still unclear whether DA influences these transcription factors and how DA interacts with the *Bm_nscaf2838_045, Bm_nscaf2674_066*, and *Bm_nscaf2767_133* genes.

Therefore, in the current experiment, the silkworm cell line Bm12 was used to survey the expression levels of *Bm_nscaf2838_045, Bm_nscaf2674_066*, and *Bm_nscaf2767_133* genes after the cells were treated with DA and the silence of transcription factors *BmRelish1*, *BmRelish2*, and *BmRel*, thereby validating the relations of the three genes with an insect’s immunity system and improving the understand of mechanism of destruxins.

## 2. Results and Conclusions

### 2.1. The Gene Expression Levels of Transcription Factors after Treatment by DA and Specific siRNA

**Figure 1:**
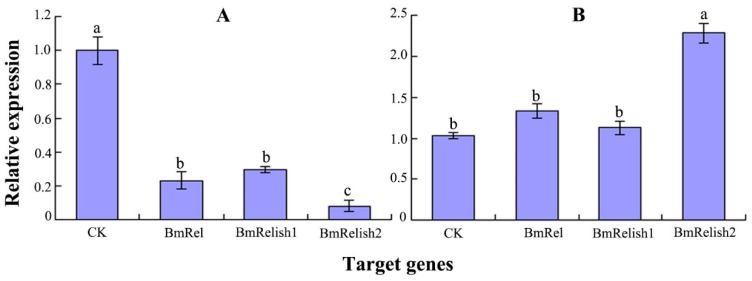
The gene expression levels of transcription factors after specific siRNA or DA treatments ((**A**) The gene expression levels after respectively treated with specific siRNA for 24 h; (**B**) The gene expression levels after 8 h of DA treatment; CK: control). The different letters on the columns indicate the significant difference (*p* < 0.05) by DMRT.

The Bm12 cells were transfected by siRNAs. After a treatment of 24 h, it was found that the transcription factors, *BmRel*, *BmRelish1*, and *BmRelish2* genes were significantly downregulated, and the interference efficiencies (relative expression) were greater than 70% (Figure 1A). Meanwhile, after 8 h of DA treatment, the expression level of the *BmRelish2* gene was significantly upregulated, but the *BmRelish1* and *BmRel* genes were basically unchanged (Figure 1B).

### 2.2. Effects of DA and Silence of BmRel/BmRelish on the Genes Expressions

**Figure 2:**
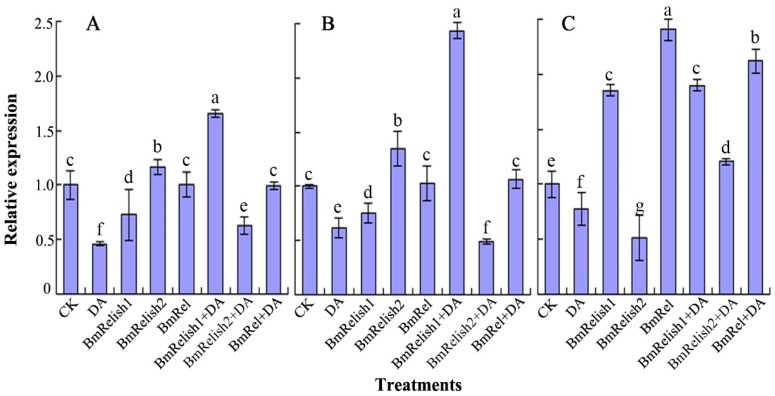
Effect of DA and transcription factor RNAi on the gene expression profiles of *Bm_nscaf2838_045* (**A**); *Bm_nscaf2674_066* (**B**); *Bm_nscaf2767_133* (**C**) (CK: control; DA: destruxin A treatment; BmRelish1/2: RNAi *BmRlish1/2* gene; BmRel: RNAi *BmRel* gene; BmRelish1/2+DA: RNAi *BmRlish1/2* gene combined with DA treatment; BmRel+DA: RNAi *BmRel* gene combined with DA treatment). The different letters on the columns indicate the significant difference (*p* < 0.05) by DMRT.

The results indicated that *Bm_nscaf2838_045*, *Bm_nscaf2674_066,* and *Bm_nscaf2767_133* genes were all downregulated after 8 h of DA treatment (Figure 2). It was also found that *Bm_nscaf2838_045* and *Bm_nscaf2674_066* genes were significantly downregulated after the *BmRelish1* gene was silenced and were upregulated when *BmRelish2* was silenced, but they were not changed after *BmRel* was silenced (Figure 2A,B). Differently, *Bm_nscaf2767_133* gene was significantly downregulated after the *BmRelish2* gene was silenced, and was significantly upregulated while *BmRel* and *BmRelish1* genes were silenced (Figure 2C).

Furthermore, when the silence of the *BmRelish1* gene and DA treatment were combined, *Bm_nscaf2838_045* and *Bm_nscaf2674_066* genes were significantly upregulated (Figure 2A,B). On the contrary, when the silence of the *BmRelish2* gene and DA treatment were combined, the two genes were all significantly downregulated. However, after the silence of the *BmRel* gene was combined with DA treatment, the two gene expressions were not statistically different from the control (Figure 2A,B). For the *Bm_nscaf2767_133* gene, the expressions were all upregulated in the treatments, except for the silence of *BmRelish2* (Figure 2C).

### 2.3. Discussion

In the current experiment, after 8 h of DA treatment, the expression level of *BmRelish2* transcript was significantly upregulated, but the *BmRelish1* transcript was basically unchanged. The results suggest that DA probably promotes the formation of the *BmRelish2* transcript prior to the *BmRelish1* transcript in the mRNA splicing of the *BmRelish* gene.

Additional data indicated that the expressions of *Bm_nscaf2838_045* and *Bm_nscaf2674_066* genes were significantly changed after the *BmRelish1/2* genes were silenced, respectively, but were not statistically changed when the *BmRel* gene was silenced. However, the *Bm_nscaf2767_133* gene expression in the silencing of the *BmRelish1/2* and *BmRel* genes were all changed. These results suggest the *Bm_nscaf2838_045* and *Bm_nscaf2674_066* genes are closely related to the Imd signal pathway, but the *Bm_nscaf2767_133* gene seems to involve in both the Imd and Toll pathways.

Because silencing *BmRelish1* decreased the gene expression levels of *Bm_nscaf2838_045* and *Bm_nscaf2674_066*, but silencing *BmRelish2* increased the two gene expressions. It is suggested that *BmRelish1* acts as an activator, while *BmRelish2* acts as a repressor for both *Bm_nscaf2838_045* and *Bm_nscaf2674_066* gene expressions.

Furthermore, the expression levels of the *Bm_nscaf2838_045* and *Bm_nscaf2674_066* genes were improved when *BmRelish1* silencing and DA treatment were combined, and both genes were downregulated after *BmRelish2* silencing was combined with DA treatment. The results support the hypothesis that DA promotes the formation of the *BmRelish2* transcript and that *BmRelish1* acts as an activator, while *BmRelish2* acts as a repressor for the two gene expressions.

Although the involvement of the three genes in an insect’s immunity was verified in the current study, few of their functions are known. In general, the transcription factors of *Rel* and *Relish* regulate the biosynthesis of antimicrobial peptides. However, the three genes do not encode antimicrobial peptides. In fact, from the literatures of gene and protein data banks, there are some information regarding these genes and functions.

The *Bm_nscaf2838_045* gene with 546 bp is predicted to encode an actin cytoskeleton-regulatory complex protein PAN1-like [*Bombyx mori*] through NCBI Blast. This protein contains the PRK15313 CDD and has the functions as a component of the PAN1 actin cytoskeleton-regulatory complex required for the internalization of endosomes during actin-coupled endocytosis. The complex links the site of endocytosis to the cell membrane-associated actin cytoskeleton, mediates uptake of external molecules and vacuolar degradation of plasma membrane proteins, plays a role in the proper organization of the cell membrane-associated actin cytoskeleton, and promotes its destabilization. It is also required for the bipolar budding of diploid cells and the correct distribution of chitin at the cell surface [13,14,15].

The *Bm_nscaf2674_066* gene encodes a serine protease (SP). It was found that SPs possess species specificity and 143 SP genes were identified in silkworm [16]. SPs are enzymes that cleave peptide bonds in proteins in which serine serves as the nucleophilic amino acid at the (enzyme’s) active site, so SPs are called serine endopeptidases. They are found ubiquitously in both eukaryotes and prokaryotes with the two broad categories based on their structure: chymotrypsin-like (trypsin-like) or subtilisin-like. SPs have multiple functions including digestion, immune response, blood coagulation, and reproduction [17,18]. SPs are involved in silkworm’s immune response against *Escherichia coli*, *Beauveria bassiana*, and the *Bombyx mori* nuclear polyhydrosis virus. SPs play an important role in the extracellular signal cascade of the immune response associated with the Toll pathway [19].

According to some data, the *Bm_nscaf2767_133* gene with 558 bp is predicted to encode a classical arabinogalactan protein 9-like. This protein is usually found in plants and seems to be implicated in diverse developmental roles such as differentiation, cell–cell recognition, embryogenesis, and programmed cell death. The protein contains a domain rich in hydroxyproline/proline, serine, alanine, and glycine amino acids. The protein has repeated glycomodules (Alanine/Serine/Threonine-Proline) and hydroxyproline, which supply the sites for O-linked glycosylation and arabinogalactan modification [20].

## 3. Materials and Methods

### 3.1. Cells and Culture

The silkworm Bm12 cell line donated by Professor Cao Yang (College of Animal Science in South China Agricultural University) was cultured with a TNM-FH culture medium (Hyclone^TM^, GE Healthcare, Pittsburgh, PA, USA), and 10% fetal bovine serum (Gibco^TM^, Thermo Fisher Scientific, Waltham, MA, USA) was added at a constant temperature culture and passage under 27 °C. Over a period of 2–4 days, the cells in logarithmic phase were used for the experiment.

### 3.2. Destruxin A and Treatment

Destruxin A (DA) was isolated and purified from the *Metarhizium anisopliae* var. *anisopliae* strain MaQ10 in the laboratory [21]. DA stock solution of 10,000 μg·mL^−1^ was made up of 1 mg of DA and 100 μL of dimethyl sulfoxide (DMSO, Sigma-Aldrich, Darmstadt, Germany). When treatment began, the DA stock solution was added into a cell well and maintained a final concentration of DA 200 μg·mL^−1^. The control group was only supplemented with 0.1% DMSO. The experiments were duplicated three times.

### 3.3. Gene RNAi Silence

To silence the transcription factor genes—*BmRel*, *BmRelish*1, and Bm*Relish*2—RNAi technology was employed. The small interfering RNA (siRNA) sequences were designed according to the 3’ end adjacent nucleotide sequence of the target genes and were synthetized by Invitrogen (Shanghai, China) (Table 1).

For preparation of the siRNA-lipo2000 mixture, two microliter (μL) lipofectimine2000 (Bio-Rad, Hercules, CA, USA) was first diluted with 200 μL of the serum-free TNM-FH culture medium and incubated at room temperature for 5 min. Meanwhile, 2 μL of siRNA was diluted with 200 μL of the serum-free TNM-FH culture medium. Then, the two solutions were mixed and incubated at room temperature for 20 min to form a siRNA-lipofectimine2000 mixture for the cell transfection experiment.

For cell transfection, the Bm12 cells in the Logarithmic phase were moved into 12-well plates (Corning Incorporated, Corning, NY, USA) and cultured with a TNM-FH culture medium. After 24 h, the culture medium was sucked out and supplemented with 100 μL of serum-free medium, which was then gently shaken. Then, 400 μL of siRNA-lipofectimine2000 mixture was added to each well and incubated at 27 °C. After 24 h, the supernatant was washed out, and transfection was terminated. Then, fresh TNM-FH medium containing serum was added, and incubation occurred for 20 h for the combination treatment with DA and a survey of the gene expression. The experiments were replicated three times. The control group was only treated with a 1% (*v*/*v*) solution of lipofectimine2000/serum-free TNM-FH culture medium.

### 3.4. Combination Treatment of Gene RNAi Silence and DA

For combination treatment of gene RNAi with DA, the cell wells treated with siRNA as described in Section 3.3 were supplemented with DA stock solution at a final concentration of 200 μg·mL^−1^. The control group was only supplemented with 0.1% dimethyl sulfoxide. The experiments were repeated three times.

### 3.5. Survey of Gene Expression

Real-time quantitative PCR (qPCR) was used to detect the gene expressions. First, total RNA was extracted from the cells treated as described above. The qPCR method refers to the Livak literature [22] and carried out by employing the CFX Connect Real-Time System (BIO-RAD, Shanghai, China). The PCR reaction system was 1 μL of cDNA of reverse transcription, upstream and downstream primers of 1 μL each (Table 2), 10 μL of SYBR Premix Ex TaqTM (Bioscience, Shanghai, China), and 7 μL of ddH_2_O. The qPCR reactive program was subjected to 39 cycles at 95 °C for 10 s, 60 °C for 10 s, 72 °C for 30 s, then 95 °C for 10 s, and 65–95 °C for 5 s. The experiment was repeated three times. The silkworm *GAPDH* (glyceraldehyde-3-phosphate dehydrogenase) gene was taken as the reference gene.

### 3.6. Data Analysis

The qPCR data were analyzed by using the 2^−ΔΔCt^ method [23] and calculated according to formula: ΔΔCt = (Ct _target gene_ − Ct _housekeeping gene_) _experimental group_ − (Ct_target gene_ − Ct_housekeeping gene_) _control group_. The relative expression of the target genes in the experimental treatment group (Q) was Q = 2^−ΔΔCt^. The means and DMRT (Duncan multiple range test) were evaluated by employing SPSS software (IBM, Armonk, NY, USA).

## 4. Conclusions

In conclusion, the *Bm_nscaf2838_045* and *Bm_nscaf2674_066* genes have similar performances. They were downregulated with individual *BmRelish1* gene silence or DA treatment, and upregulated with *BmRelish1* gene silence combined with DA treatment. Furthermore, the two genes were upregulated with individual *BmRelish2* gene silence and downregulated with combination of *BmRelish2* gene silence plus DA treatment, but their expression levels were not changed in *BmRel* gene silence combined with DA treatment. However, for the *Bm_nscaf2767_133* gene, downregulated expression was found in individual *BmRelish2* gene silence or DA treatment, and upregulated expressions were found in the combination of *BmRelish2* gene silence and DA treatment, and in the individual *BmRel* or *BmRelish1* silence. The results suggest that the gene expressions of *Bm_nscaf2838_045* and *Bm_nscaf2674_066* are closely related to the Imd signal pathway, but the *Bm_nscaf2767_133* might be involved in both the Imd and Toll pathways. Furthermore, for both *Bm_nscaf2838_045* and *Bm_nscaf2674_066* gene expressions, the *BmRelish1* may act as an activator, while *BmRelish2* probably acts as a repressor. Meanwhile, the research results imply that DA may promote splicing *BmRelish* into the *BmRelish2* as opposed to *BmRelish1.* Our research will provide new insight on the understanding of the activity mechanisms of destruxins.

## Figures and Tables

**Table 1:** Small interfering RNA (siRNA) for silencing target genes.

Target Gene	siRNA Sequence
*BmRel*	5’-GCAAACGAGACGAGACCUUTT-3’
	3’-TTCGUUUGCUCUGCUCUGGAA-5’
*BmRelish1*	5’-GCAGUUCCCGAAUCUGCAATT-3’
	3’-TTCGUCAAGGGCUUAGACGUU-5’
*BmRelish2*	5’-CCCAUUGAAAUGACUUGAATT-3’
	3’-TTGGGUAACUUUACUGAACUU-5’

**Table 2:** Primers of the silkworm genes for qPCR detecting.

Gene	Primer (5’→3’)
*BmRelish*1	F: CCTGGAAAATGTCTGCCGATAA
	R: ATGCCGTCTAGTGCCGTGCT
*BmRelish*2	F: AGCAGTTATGCGTTTCGGTTTG
	R: AATGCTGCCACCCACCTTG
*BmRel*	F: AATGACCCCAATCAACCTAACG
	R: CGGAATCTGAGGGCTTTGC
*Bm_nscaf2838_045* (GeneBank access NO. KY379951)	F: CCTGCCATTGTTGAAGGTGC
	R: GAGGGTCTCGGGAAGAGTGA
*Bm_nscaf2674_066* (GeneBank access NO. KY379952)	F: GTGTATCTGACGGCAACCTC
	R: ATGTCATTGGCATTGCTCTT
*Bm_nscaf2767_133* (GeneBank access NO. KY379953)	F: GTTGAGGACATTGCCGAAGA
	R: ACCAACAATTACGGGCTTGA
*GAPDH* (reference gene)	F: ATGTTTGTTGTGGGTGTTA
	R:GTAGAGGCAGGAATGATT
